# Mesenchymal Stem/Stromal Cells in Immunity and Disease: A Better Understanding for an Improved Use

**DOI:** 10.3390/jcm9051516

**Published:** 2020-05-18

**Authors:** Makram Merimi, Laurence Lagneaux, Douâa Moussa Agha, Philippe Lewalle, Nathalie Meuleman, Arsène Burny, Hassan Fahmi, Mehdi Najar

**Affiliations:** 1 Laboratory of Experimental Hematology, Institut Jules Bordet, Université Libre de Bruxelles (ULB), 1000 Bruxelles, Belgium; makram.merimi.cri@gmail.com (M.M.); douaa.moussa@gmail.com (D.M.A.); philippe.lewalle@bordet.be (P.L.); nathalie.meuleman@bordet.be (N.M.); 2 Laboratory of Physiology, Genetics and Ethnopharmacology, Faculty of Sciences, University Mohammed Premier, 60000 Oujda, Morocco; 3 Laboratory of Clinical Cell Therapy, Institut Jules Bordet, Université Libre de Bruxelles (ULB), 1070 Brussels, Belgium; laurence.lagneaux@bordet.be; 4 Laboratory of Molecular and Cellular Biology, Faculté Universitaire des Sciences Agronomiques de Gembloux (FUSAGx), 5030 Gembloux, Belgium; arsene.burny@guest.uliege.be; 5 Osteoarthritis Research Unit, University of Montreal Hospital Research Center (CRCHUM), Montreal, QC H2X 0A9, Canada; h.fahmi@umontreal.ca

**Keywords:** MSCs, therapy, immune cells, tissue environment

## Abstract

In this Special Issue, directed and supervised by Dr. Mehdi Najar, a collection of basic research articles and reviews, on the state of the art of Mesenchymal Stem/Stromal Cells (MSCs) immune biology, is presented. Among the major goals of this Special Issue is the presentation of an update about the immunomodulatory properties of MSCs and their capacity to respond to tissue microenvironment changes. MSCs hold great promise in the field of immunotherapy and regenerative medicine. Accordingly, a better understanding of MSC immune biology will improve their therapeutic value and use.

Mesenchymal stem/stromal cells (MSCs) have been described as multipotent fibroblast-like cells that can be virtually found in almost all tissues. Due to their simple and easy isolation procedure, they can be expanded by in vitro cultures, and therefore used for different clinical applications [1]. The therapeutic effect of MSCs is attributed to complex cellular and molecular mechanisms involved in the regulation and modulation of immune responses. MSCs are not true immune cells, but medicinal signaling cells harboring several paracrine signaling molecules. Moreover, MSCs are environmentally responsive, in detecting tissue challenges and in adapting, in consequence, their behavior. In parallel, MSCs were found to carry extracellular vesicles (EVs) that mediate their immunomodulatory effects, by transferring their cytokines, messenger ribonucleic acid (mRNA), or micro ribonucleic acid (miRNA) contents to the target cells. Accordingly, their use as immunotherapeutic strategies will present new hopes for treating patients with immunological and inflammatory diseases. While preclinical data indicates that MSCs have important immunomodulatory properties, further studies are still in progress to increase the safety and efficiency of MSC-based therapy. In this Special Issue, we proposed to experts in the field to present their recent research findings, as well as their insights regarding the immuno-biology properties of MSCs. A better understanding of the mechanisms underlying the therapeutic effects of MSCs and the influence of the local environment will improve their clinical use. In that context, Laroye, C. et al. reported that both Wharton’s Jelly (WJ) and bone marrow (BM) sources allow a quick and easy clinical grade production of MSCs, with slight differences regarding identity, safety and functionality [2]. Iacobaeus, E. et al. successfully conducted a phase I clinical study using bone-marrow MSCs as a possible new therapy for Multiple Sclerosis (MS). Such therapy was safe, well tolerated, and associated with possible transient, beneficial, clinical and peripheral immune-tolerogenic effects [3]. Velier, M. et al. indicated that adipose-derived stem/stromal cells (ASC) from systemic sclerosis (SSs) patients maintain pro-angiogenic and anti-fibrotic paracrine effects in vitro, thereby supporting further development of ASC-based autologous therapies for SSc treatment [4]. Buyl, K. et al. pointed out the responses and characteristics of MSCs from adipose tissue, following expansion and inflammatory priming that substantially modulated the expression of 28 molecules implicated in immune regulation [5]. Consequently, Voisin, C. et al. indicated that MSCs from the umbilical cord remained hypoimmunogenic, and retained their immunomodulatory properties during chondrocyte differentiation [6]. Further, Elango, J. et al. noted that the parathyroid hormone-related protein (PTHrP) shows accelerated soluble receptor activator of nuclear factor (NF)-kB ligand (sRANKL) downregulation of osteogenesis of MSCs, by increasing RANK expression at the earlier stage of differentiation and by inhibiting ANK [7]. On the other hand, Ising, R. et al. successfully differentiated cord blood-derived hematopoietic progenitor into mature CD56+CD94+NKG2A+NK (Natural Killer) cells on HCMV (Human cytomegalovirus)-infected MSCs from bone marrow, with significantly higher anti-viral cytokine production compared to NK cells developing on non-infected MSCs [8]. Behm, C. et al. observed that Vitamin D3 increases or decreases the immunomodulatory activities of Periodontal ligament-MSCs, depending qualitatively and quantitatively on the presence of specific tumor necrosis factors TNF-α-, Interleukin IL-1β-, and Interferon IFN-γ inflammatory cytokines [9]. Zarriello, S. et al. suggest that Bone marrow mesenchymal stem/stromal cells BM-MSCs containing Tregs are more therapeutically active, because they present an increased stem cell activity after stroke-induced hypoxia [10]. 

As a review, Behnke, J. et al. discussed the most recent advances in MSC-based therapy, and highlighted joint mechanisms of MSC action across disease entities, which provides the basis to timely tackle this global disease burden [11]. The review of Crippa, S. et al. revised the role of MSCs in the orchestration of the BM niche, and discussed possible alterations in the mesenchymal compartment in specific disorders. They focused on the need to correct and restore a proper microenvironment to ameliorate transplantation procedures, and more general disease outcomes [12]. Batsali, A.K. et al. summarized the role and therapeutic potential of MSC-EVs (Extracellular vesicles) in normal and malignant hematopoiesis, as well as their potential contribution in treating Graft versus host disease (GVHD) [13]. Sava, R.I. et al. reviewed how MSCs efficiently target immune dysregulation in Heart failure with preserved ejection fraction (HFpEF) and stop disease progression. In vitro, MSCs may reduce the pro-inflammatory activity of immune cell types, and reduce myocardial fibrosis and improve diastolic function in vivo [14]. Zhou, Y. et al. presented the recent advances regarding the immunomodulatory mechanisms of MSCs, and the importance of their paracrine factors in regenerative medicine and immune diseases [15]. In line with this, Alfaro D. et al. provided new results on the effects of the Eph/ephrins pathway in the differentiation and immunomodulatory properties of MSCs [16]. Collectively, these studies allowed a better understanding of the immune-biology of MSCs in normal and diseased conditions, and, therefore, will contribute to improving their therapeutic effects for future clinical applications.
